# Views from the Coalface: What Do English Stop Smoking Service Personnel Think about E-Cigarettes?

**DOI:** 10.3390/ijerph121215048

**Published:** 2015-12-21

**Authors:** Rosemary Hiscock, Linda Bauld, Deborah Arnott, Martin Dockrell, Louise Ross, Andy McEwen

**Affiliations:** 1 Department of Health, University of Bath, Bath BA2 7AY, UK; 2 UK Centre for Tobacco and Alcohol Studies (UKCTAS), Nottingham NG5 1PB, UK; linda.bauld@stir.ac.uk (L.B.); andy.mcewen@ncsct.co.uk (A.M.); 3 School of Health Sciences, University of Stirling, Stirling FK9 4LA, UK; 4 Action on Smoking and Health (ASH), Suites 59–63, 6th Floor, New House, 67–68 Hatton Garden, London EC1N 8JY, UK; deborah.arnott@ash.org.uk; 5 Public Health England, Skipton House, 80 London Road, London, SE1 6LH, UK; Martin.Dockrell@phe.gov.uk; 6 Stop Smoking Service Leicester City Council, Leicester LE1 6TH, UK; louise.ross@leicester.gov.uk; 7 National Centre for Smoking Cessation and Training (NCSCT), 1-6 Yarmouth Place, LondonW1J 7BU, UK

**Keywords:** e-cigarettes, stop smoking services, cessation, harm reduction

## Abstract

The UK Stop Smoking Services (SSS) are a source of information and advice on e-cigarettes for smokers and thus it is important to understand the knowledge of, and attitudes towards, e-cigarettes held by stop smoking practitioners. The datasets were English SSS quarterly monitoring returns (*n* = 207,883) and an online survey of English SSS practitioners, managers, and commissioners between 26th November and 15th December 2014 (*n* = 1801). SSS monitoring data suggested 2% of clients were using e-cigarettes to quit with SSS and that clients using e-cigarettes had similar quit rates to clients using Varenicline. Most SSS personnel are waiting for licenced e-cigarettes to become available before they will recommend them to clients. However, less than a quarter view e-cigarettes as “a good thing”. Managers and commissioners were more positive than practitioners. SSS personnel working for the NHS (hospitals and GP surgeries) were less positive about e-cigarettes than those employed elsewhere. E-cigarettes were cited as the most important reason for the recent decline in service footfall. Thus dissemination of information about e-cigarettes needs to be examined and services should address their stance on e-cigarettes with some urgency.

## 1. Introduction

E-cigarettes usually provide nicotine and potentially act as a replacement for smoking. They are battery powered devices that aim to simulate tobacco cigarettes, essentially by heating a solution containing nicotine into an inhalable aerosol [1,2]. Available evidence, so far, suggests that e-cigarettes are much safer than tobacco because the constituents of cigarette smoke that are harmful are either absent or present at much lower levels in e-cigarettes and the chemicals present in e-cigarettes have not, at least as yet, been shown to be harmful [3,4,5]. They have also not become a significant gateway to smoking cigarettes for non-smokers [4]. Furthermore at least some types of e-cigarettes might help smokers to quit or at least reduce their cigarette use [3,5,6]. Meta-analysis of randomised control trials [7] has shown that e-cigarettes are similar in efficacy to patches for quitting and are better than patches for reducing the number of cigarettes smoked and quitting and harm reduction are more likely when e-cigarettes contain nicotine; however the small number of trials means that more trials are required. In 2015 there were about 2.6 million e-cigarette users in Great Britain [8]. Among a survey sample of smokers and ex-smokers in England a fifth were current users and a third had tried them [9]. E-cigarettes are now, by a considerable margin, the approach to quitting most favoured by smokers in England [10].

A small proportion of smokers (about 5% of those making a quit attempt [10]) are willing to access local stop smoking services (SSS) for help with their quit attempt, but this still amounted to 450,582 clients in 2014/2015 [11]. SSS treated 816,444 smokers in 2011/12 [11] and thus use has almost halved between 2011/12 and 2014/15. At the same time e-cigarette use has grown, however, during this period the SSS have also undergone other changes such as being transferred from the NHS to Local Authorities. Nevertheless, smokers attending SSS have a greater chance of quitting than smokers attempting to stop by using medication or willpower alone [12,13,14] and a growing number of countries are offering cessation support [15]. Thus the reported experience of smokers already willing to use SSS, a cessation aid which has empirical support [16], regarding e-cigarettes may be particularly valuable. 

The National Centre for Smoking Cessation Training (NCSCT) recommends that SSS “be open to electronic cigarette use in people keen to try them; especially in those that have tried, but not succeeded, in stopping smoking with the use of licensed stop smoking medicines” [17]. However the safety of e-cigarettes is still debated [18] and the ensuing confusion among potential consumers may be the reason for the current plateauing of growth [19,20]. Previous research suggests that SSS are a source of information and advice on e-cigarettes for smokers [21] and as such it is important to understand the knowledge of, and attitudes towards, e-cigarettes held by stop smoking practitioners. 

The aims of this paper are to explore:

- Reported extent and efficacy of e-cigarette use among Stop Smoking Service clients
- SSS personnel’s knowledge and attitudes towards e-cigarettes and which groups are more likely to be positive about e-cigarettes
- Whether SSS personnel view e-cigarettes as having an impact on the recent reduction in SSS use by smokers wishing to quit

## 2. Experimental Section

### 2.1. Data

#### 2.1.1. Local Stop Smoking Services Quarterly Returns

Quarterly monitoring returns of the local stop smoking services data (April to June and July to September 2014) were analysed. Four times a year, SSS send data on the number of clients who have set a quit date (named a day when they will attempt to quit smoking) and how many of these had quit smoking four weeks later to the NHS Health and Social Care Information Centre. Clients can be counted in national monitoring data if they are an adult who has been smoking a tobacco product daily or an adolescent who has been smoking a tobacco product weekly and they have not quit smoking more than 48 hours prior to attending their first session [12]. A client is regarded as a four week quitter if they self-report that they have not smoked within the previous two weeks and an expired air carbon monoxide test reading is 10ppm or below [12]. The information is originally collected by each SSS client’s practitioner either electronically using data monitoring software or via paper forms. Since 1st April 2013, this data collation has not been mandatory but the majority of services continue to send information [12]. 

From 1st April 2014, services were asked to include whether SSS clients were using e-cigarettes and similar products, such as nicotine strips for oral use, which together were called “unlicensed nicotine containing products” in addition to continuing to record if they were using licensed medications (nicotine replacement therapy (NRT), Bupropion or Varenicline) [12]. Note that, currently, e-cigarettes account for virtually all unlicensed nicotine containing products. Clients could also use different combinations of licenced and unlicensed products and these are also recorded.

#### 2.1.2. Online Survey

An e-bulletin was sent to 24,000 SSS practitioners, managers, and commissioners registered with the NCSCT containing an invitation to participate and a link to an online survey. Practitioners directly provide behavioural support for smokers wanting to quit. Managers may have direct contact with smokers through supervising practitioners. Commissioners work for the local authority and make decisions about who will run the service and are less likely to have direct contact with clients. The survey was completed 1801 times between 26 November and 15 December 2014. After exclusions for missing data and locations outside England there were 1761 cases available for analysis. This represents a 7% response rate or one in every 14 of those invited.

### 2.2. Analysis

The software used for analysis was SPSS version 22. The data collected in the online survey will be deposited with the University of Bath Research Archive.

#### 2.2.1. Local Stop Smoking Services Quarterly Returns

Frequencies and four-week quit rates were calculated by the nicotine containing product (NCP) used. The categories available were single licensed NCP only, combination of licensed NCPs concurrently, Bupropion (Zyban) only, Varenicline (Champix) only, licensed NCP and/ or Bupropion (Zyban) and/or Varenicline (Champix) consecutively, combination of a licensed medication and an unlicensed NCP concurrently, licensed medication and an unlicensed NCP consecutively, unlicensed NCP only, did not use any licensed medication, or unlicensed NCP, not known. Additionally, the total setting quit dates and quitting for clients using an unlicensed NCP alone or in combination with other medications was calculated. Clients who are lost to follow up are included in the denominator in an “intention to treat” approach [22].

#### 2.2.2. Online Survey

Univariate results and column percentages were tabulated. For most questions, respondents were asked to tick as many as applied and for these the number and proportion that ticked each box are tabulated. For some questions, clients were asked whether they agreed with a list of statements and answered on a five point Likert scale. For analysis, these were dichotomised into strongly agree and agree and compared with neither agree nor disagree, disagree, and strongly disagree. 

Opinion of e-cigarettes was the dependent variable for most of the analysis. Opinion of e-cigarettes was derived from responses to how much respondents agreed with the statement that e-cigarettes are a good thing. Respondents who held a positive opinion of e-cigarettes if they said they strongly agreed or agreed were compared with respondents who said they neither agreed nor disagreed, disagreed, or strongly disagreed. Chi-square tests, with the continuity correction applied for two by two tables, were conducted between opinion of e-cigarettes and sources of information on e-cigarettes, (possible sources were NCSCT briefing and website, the news and media, training courses, ASH briefing and website, WHO report on electronic cigarettes, experienced electronic cigarette users, the websites of electronic cigarette companies, the websites of vaping organisations); whether respondents agreed or strongly agreed (compared with neither agreed or disagreed, disagreed or strongly disagreed) that: “e-cigarettes should not be recommended by SSS until there is good evidence on safety and effectiveness”, “if an e-cig was a licensed medication, I would definitely recommend them to clients”, “e-cigarettes should only be available as a licensed medication”, “e-cigarettes normalise cigarette smoking”, “over the past twelve months, I’ve become less positive about e-cigarettes”, “e-cigarettes are a good thing”, “e-cigarettes should be able to be bought anywhere by smokers as a consumer product” and “e-cigarettes denormalise cigarette smoking”; client groups to which respondents would recommend e-cigarettes (possible groups were all my clients, clients who are already using e-cigarettes, clients wishing to cut down but not stop, clients wanting to use electronic cigarettes at times when they cannot smoke (temporary abstinence), clients who wish to cut down before they stop, clients who have tried and failed to quit many times, more dependent smokers, none of my clients); region of England (North East, North West, Yorkshire & Humberside, East Midlands, West Midlands, East of England, London, South East, South West), employer organization (General Practice, Pharmacy, Acute/Foundation Hospital Trust, Employed directly by the Local Authority, Mental Health Trust, Private company, Social enterprise, Other) and professional role (Commissioner, Manager, Stop Smoking Practitioner, Other). Regional differences have been found for preferred tobacco product [23] and smoking prevalence [24] and area differences have been found for smoking cessation [13]. Thus regional differences for e-cigarettes were plausible and included in the analysis. Differences have also been found for cessation according to stop smoking service provider [13,25] so employer organisation was included and analysed. In preparation for multivariate analyses chi-square tests were also conducted between each of the variables and professional role in the services.

There was also univariate analyses of reasons for the decline in e-cigarette use (reasons were smokers choosing to use e-cigarettes, remaining smokers in the population are harder to reach, reduced mass media health campaigns, reduced funding of services, SSS moved from NHS to LA, SSS put out to tender and few specialist practitioners available). 

#### 2.2.3. Multivariate Analysis—Which Variables Affect the Association between Role in the Service and Opinion of E-Cigarettes

Logistic regression analysis, with opinion on e-cigarettes as the outcome, was carried out. Variables were candidates to be included in the model where they were significantly associated with opinion on e-cigarettes and role in the services in bivariate analysis. The following stages took place: Firstly, cases which were missing on any variable, or whose role in the service was “other” were excluded leaving 1278 cases. Secondly, each variable was placed alone in the model. Thirdly, each variable was placed in the model with role in the service and the change in the odds ratio of role in the service was noted. Fourthly, the variables were added in a stepwise fashion; variables were entered in the order of the size of the difference made to the odds ratio of role in the service in the second stage. When the odds ratio of role in the service was non-significant (*p* < 10) no more independent variables were entered into the model. The relative importance of the variables was checked using backwards stepwise entry.

## 3. Results and Discussion

### 3.1. Local Stop Smoking Services Quarterly Returns

Of the 207,883 clients who set quit dates between April and September 2014, 4750 reported using electronic cigarettes (Table 1). There were 874 clients who reported using electronic cigarettes only, 3122 who used them concurrently with a licenced medication and 874 who reported used them consecutively with a licenced medication (the data does not allow us to determine whether e-cigarettes were used first or second). Self report quit rates for those using electronic cigarettes (59%) were higher than general (50%) and similar to those using Varenicline alone (60%).

**Table 1:** English local Stop Smoking Services monitoring data April to September 2014.

Type of Pharmacotherapy	Set Quit Date	Quit at 4 Weeks	% Quit
Total	207,883	103,899	50%
Single NCP only	60,513	28,954	48%
Combination of licensed NCPs concurrently	62,771	28,728	46%
Bupropion (Zyban) only	1047	582	56%
Varenicline (Champix) only	53,215	32,002	60%
Licensed NCP and/or Bupropion (Zyban) and/or Varenicline (Champix) consecutively	3542	1674	47%
Combination of a licensed medication and an unlicensed NCP concurrently	3122	1756	56%
Licensed medication and an unlicensed NCP consecutively	754	464	62%
Unlicensed NCP only	874	570	65%
Did not use any licensed medication or unlicensed NCP	11,716	6314	54%
Not known	10,329	2855	28%
Unlicensed NCP with and without other medication	4750	2790	59%
% Total clients using NCP	2%	3%	

### 3.2. Online Survey

#### 3.2.1. Respondent Knowledge and Attitudes

The most frequently mentioned source of information on e-cigarettes, mentioned by about two-thirds of respondents, was the NCSCT and the second was news and media (Table 2). Over half of respondents said they would not recommend e-cigarettes to any of their clients. Contrastingly, less than 5% would recommend e-cigarettes to all their clients.

Respondents were asked whether they agreed with eight statements. Over three-quarters agreed that that e-cigarettes should not be recommended by SSS until there is good evidence on safety and effectiveness but two-thirds agreed that if they were licensed that they would recommend them and nearly 60% agreed that they should only be available as a licensed medication. Just under a fifth thought that e-cigarettes should be available anywhere as a consumer product. Only about half agreed that e-cigarettes normalize cigarette smoking although only a tenth agreed that e-cigarettes denormalise smoking. About a quarter of respondents had become less positive about e-cigarettes over the last 12 months; nevertheless about a quarter also thought that e-cigarettes were a good thing. 

The variables which were significantly associated with both role in the service and opinion of e-cigarettes and were thus candidates for entry into the multivariate analyses were the following sources of information: ASH briefing and website, WHO report on electronic cigarettes and experienced e-cigarette users; the following statements: “Over the past 12 months I’ve become less positive about e-cigarettes”, “if an e-cigarette was a licensed medication, I would definitely recommend them to clients”, and “e-cigarettes denormalise cigarette smoking” and the final variable was whether respondents would recommend clients wanting to use electronic cigarettes at times when they cannot smoke (temporary abstinence).

#### 3.2.2. Spread of Service Providers Stances on E-Cigarettes (Bivariate Analyses)

The largest regional representation was from London and the southeast and the smallest was from the northeast (Table 3). Respondents working in London made up a higher proportion of those positive about e-cigarettes compared with those in the north of England.

Nearly half of respondents were employed by GP practices or pharmacies whereas managers/commissioners were most likely to be employed by a hospital trust or directly by a local authority. Those employed by GPs and hospitals were less likely to hold a positive opinion of e-cigarettes whereas those employed by mental health trusts or local authorities were more positive. Nearly two-thirds of respondents were practitioners, 8% were managers, and 1% were commissioners. Commissioners and managers made a larger than expected proportion of those positive about e-cigarettes whereas practitioners made up a smaller than expected proportion.

**Table 2:** Respondent knowledge and attitudes by role in services and opinion on e-cigarettes.

Variable	Distribution		Role in Services					Opinion on E-Cigs				
			Manager/Commissioner		Practitioner		Sig.	Agree “A Good Thing”		Neutral/Disagree		Sig.
	N	%	N	%	N	%		N	%	N	%	
*Sources of information on e-cigs*												
NCSCT briefing and website	1155	65.6	126	78.8	821	68.2	*p* = 0.009	281	65.5	863	65.9	*p* = 0.933
The news and media	797	45.3	74	46.3	549	45.6	*p* = 0.951	203	47.3	586	44.7	*p* = 0.380
Training courses	670	38.0	56	35.0	496	41.2	*p* = 0.155	165	38.5	501	38.2	*p* = 0.982
ASH briefing and website	585	33.2	109	68.1	383	31.8	*p* < 0.001	173	40.3	407	31.1	*p* = 0.001
WHO report on electronic cigarettes	517	29.4	74	46.3	320	26.6	*p* < 0.001	143	33.3	369	28.2	*p* = 0.048
Experienced electronic cigarette users	457	26.0	54	33.8	302	25.1	*p* = 0.025	169	39.4	285	21.8	*p* < 0.001
The websites of electronic cigarette companies	189	10.7	20	12.5	122	10.1	*p* = 0.436	61	14.2	127	9.7	*p* = 0.011
The websites of vaping organisations	114	6.5	26	16.3	63	5.2	*p* < 0.001	39	9.1	74	5.6	*p* = 0.017
*Respondents agreed or strongly agreed (compared with neither agreed or disagreed, disagreed or strongly disagreed) that:*												
E-cigs are a good thing?	429	24.4	60	38.0	264	22.2	*p* < 0.001					
Over the past 12 months I've become less positive about e-cigs	495	28.1	31	19.5	362	30.7	*p* = 0.005	34	8.0	457	35.2	*p* < 0.001
E-cigs should not be recommended by SSS until there is good evidence on safety & effectiveness	1342	76.2	118	74.7	944	79.7	*p* = 0.180	234	54.9	1101	84.9	*p* < 0.001
If an e-cig was a licensed medication, I would definitely recommend them to clients	1148	65.2	121	76.6	790	66.5	*p* = 0.014	375	88.0	765	58.9	*p* < 0.001
E-cigs should only be available as a licensed medication	1036	58.8	97	61.0	752	63.5	*p* = 0.597	164	38.7	863	66.6	*p* < 0.001
E-cigs should be able to be bought anywhere by smokers as a consumer product	325	18.5	34	21.7	203	17.2	*p* = 0.202	193	45.7	130	10.0	*p* < 0.001
E-cigs normalise cigarette smoking	950	53.9	88	55.7	670	57.2	*p* = 0.791	140	33.3	804	62.4	*p* < 0.001
E-cigs denormalise cigarette smoking	190	10.8	29	19.5	118	10.3	*p* = 0.001	66	15.9	124	9.9	*p* = 0.001
*Client groups to which respondents would recommend e-cigarettes*												
All my clients	82	4.7	10	6.3	45	3.7	*p* = 0.193	58	13.5	23	1.8	*p* < 0.001
Clients who are already using e-cigs	324	18.4	40	25.0	219	18.2	*p* = 0.051	138	32.2	184	14.0	*p* < 0.001
Clients wishing to cut down but not stop	215	12.2	24	15.0	139	11.6	*p* = 0.258	104	24.2	111	8.5	*p* < 0.001
Clients wanting to use electronic cigarettes at times when they cannot smoke (temporary abstinence)	212	12.0	31	19.4	131	10.9	*p* = 0.003	105	24.5	107	8.2	*p* < 0.001
Clients who wish to cut down before they stop	204	11.6	22	13.8	129	10.7	*p* = 0.312	101	23.5	102	7.8	*p* < 0.001
Clients who have tried and failed to quit many times	355	20.2	34	21.3	236	19.6	*p* = 0.703	167	38.9	187	14.3	*p* < 0.001
More dependent smokers	199	11.3	23	14.4	118	9.8	*p* = 0.100	98	22.8	100	7.6	*p* < 0.001
None of my clients	983	55.8	82	51.2	693	57.6	*p* = 0.150	122	28.4	850	64.9	*p* < 0.001
												

**Table 3:** Stances of service providers on e-cigarettes.

Variable			Role in Services				Opinion of E-Cigs			
	Total		Manager/Commissioner		Practitioner		Agree “A Good Thing”		Neutral/Disagree	
	N	%	N	%	N	%	N	%	N	%
*Region*				*p* = 0.026				*p* = 0.015		
North East	64	3.6	5	10.4	43	89.6	10	15.6	54	84.4
North West	230	13.1	23	12.6	159	87.4	47	20.6	181	79.4
Yorkshire & Humberside	132	7.5	7	7.0	93	93.0	24	18.5	106	81.5
East Midlands	159	9.0	6	5.1	112	94.9	40	25.2	119	74.8
West Midlands	224	12.7	19	10.8	157	89.2	50	22.9	168	77.1
East of England	151	8.6	14	12.5	98	87.5	35	23.5	114	76.5
London	290	16.5	32	14.5	189	85.5	94	32.6	194	67.4
South East	303	17.2	40	16.9	196	83.1	81	27.1	218	72.9
South West	208	11.8	14	8.2	156	91.8	48	23.5	156	76.5
Total	1761	100.0	160	11.7	1203	88.3	429	24.7	1310	75.3
*Organisation*				*p* < 0.001				*p* < 0.001		
General Practice	477	27.1	5	1.3	377	98.7	78	16.6	393	83.4
Pharmacy	334	19.0	25	8.9	257	91.1	83	25.2	247	74.8
Acute / Foundation Hospital Trust	255	14.5	29	16.3	149	83.7	47	18.8	203	81.2
Employed directly by the Local Authority	163	9.3	33	24.6	101	75.4	53	32.9	108	67.1
Mental Health Trust	105	6.0	16	21.3	59	78.7	45	43.7	58	56.3
Private company	104	5.9	15	18.8	65	81.3	36	35.0	67	65.0
Social enterprise	56	3.2	8	16.0	42	84.0	16	28.6	40	71.4
Other	267	15.2	29	15.9	153	84.1	71	26.8	194	73.2
Total	1761	100.0	160	11.7	1203	88.3	429	24.7	1310	75.3
*Role*								*p* < 0.001		
Commissioner	23	1.3					14	63.6	8	36.4
Manager	137	7.8					46	33.8	90	66.2
Other	398	22.6					105	26.9	286	73.1
Stop Smoking Practitioner	1203	68.3					264	22.2	926	77.8
Total	1761	100.0					429	24.7	1310	75.3

Both region and organisation were significantly associated with the role the respondent played in the services and their opinion of e-cigarettes. Thus both these variables were put forward for the multivariate analyses.

#### 3.2.3. Why might Commissioners and Managers be more Positive about E-Cigarettes than Practitioners? (Multivariate Analysis)

There were 1278 cases available for multivariate analysis out of the 1363 clients who indicated they were a practitioner, manager, or commissioner (Table 4). Managers/commissioners were approximately twice as likely to agree/strongly agree that e-cigarettes were a good thing as commissioners (OR 2.15 (1.50 to 3.08)) when role was alone in the model (Table 4). The variable that caused the largest decline in the odds ratio of role when it was entered individually was the employing organisation followed by agreement that the respondent had become less positive in the last year. 

When the variables were added in a forward stepwise manner, role became non-significant (*p* < 0.05) when three variables were added and non-significant (*p* < 0.10) when four variables were added. Thus there was no generalizable difference in opinion on e-cigarettes of practitioners and managers/commissioners once these four variables had been taken into account. Removing each of the four variables individually confirmed the order of magnitude difference made to the odds ratio of role. The most important variable was employing organisation followed by agreement of becoming less positive in the last year, then recommendation for temporary abstinence, and finally agreement that they would recommend e-cigarettes if they were licenced. 

In summary, managers/commissioners were more positive about e-cigarettes than practitioners; they were also more likely to work directly for local authorities rather than GP practices, they were less likely to say they had become less positive about e-cigarettes in the last year and they were more likely to say that they would recommend them for temporary abstinence and that they would recommend them to clients if they were licenced.

Items that did not appear to be mediators included information source about e-cigarettes, the region worked in, whether or not e-cigarettes denormalise smoking and client groups to which they would recommend cigarettes (other than temporary abstinence).

**Table 4:** Reasons for manager/commissioners more positive view of e-cigarettes (strongly agree/agree that e-cigarettes are a good thing compared with neutral, disagree, or strongly disagree) rather than practitioners suggested from logistic regression models—final mediating variables.

Variable	aOR of Manager/Practitioner When Variables Added in Forward Stepwise	Significance of Role	aOR of Manager/Practitioner When Variables Removed from Full Model Individually (Backwards Stepwise)
Role (manager/commissioner *vs* practitioner) only	2.15 (1.50 to 3.08)		
Organisation	1.82 (1.24 to 2.65)	*p* = 0.002	1.60 (1.07 to 2.37)
Agree less positive in the last year	1.59 (1.07 to 2.36)	*p* = 0.022	1.57 (1.05 to 2.35)
Recommend e-cigs for temporary abstinence	1.47 (0.98 to 2.20)	*p* = 0.064	1.49 (0.99 to 2.23)
Agree would recommend if licenced	1.39 (0.91 to 2.10) ^1^	*p* = 0.124	1.47 (0.98 to 2.20)

^1^ This was the adjusted odds ratio (aOR) of role in the final model. In the final model respondents who were employed by mental health trusts and employed directly by local authorities were significantly more likely to have a good opinion of e-cigarettes than those employed by GP practices (aOR 3.41 (1.82 to 6.37) and 1.86 (1.12 to 3.12) respectively), respondents who agreed that they had become less positive in the last year were significantly less likely to have a good opinion of e-cigarettes generally (aOR 0.16 (0.11 to 0.26)), respondents who would recommend e-cigarettes for temporary abstinence or if they were licenced were significantly more likely to have a good opinion of e-cigarettes (aOR 2.78 (1.89 to 4.07 and aOR 3.93 (2.68 to 5.77) respectively).

#### 3.2.4. Role of E-Cigarettes in Decline in SSS Uptake

E-cigarettes were seen as the most important reason for the decline in numbers attending the SSS followed by remaining smokers being harder to reach (Table 5). Thus the substantial changes in the structure and funding of SSS were less commonly cited as reasons for the decline.

**Table 5:** Reasons for the decline in SSS client numbers.

Reason	N	%
Smokers choosing to use e-cigs	1461	83.0
Remaining smokers in the population are harder to reach	947	53.8
Reduced mass media health campaigns	527	29.9
Reduced funding of services	488	27.7
SSS moved from NHS to LA	389	22.1
Put out to tender	299	17.0
Few specialist practitioners available	243	13.8

## 4. Conclusions

E-cigarettes were reported as being used by a very small proportion of SSS clients, but those clients were among the most successful. Nevertheless, most SSS personnel were waiting for licenced e-cigarettes to become available before recommending them to clients and less than a quarter viewed e-cigarettes as “a good thing”. This is probably because evidence of effectiveness has only been recently acquired [4]. A vaping promotion organisation survey of 84 medical professionals [26] found generally positive opinions on e-cigarettes but also some ambivalence; these findings have not been peer reviewed.

Managers and commissioners were more positive about e-cigarettes than practitioners. Multivariate analysis in our study suggested that reasons for the association between role and positive feelings towards e-cigarettes might include that, firstly, practitioners were more likely to work for GP practices where staff were generally more negative whereas managers/commissioners were more likely to work directly for a local authority or mental health trust where staff were generally more positive. Secondly, practitioners were more likely to say they have become less positive in the last 12 months than personnel in other roles. Thirdly, managers and commissioners were more likely to recommend e-cigarettes for temporary abstinence than practitioners—this could be due to a wider role in tobacco control and links to networks disseminating evidence and guidance on e-cigarettes and more time to peruse new research whereas practitioners may be more focussed on their cessation role. Such differences perhaps reflect lack of dissemination of information which could be addressed by services. Innovation and improvement are most likely when there is greater sharing of information [27]. SSS personnel cited e-cigarettes as the most important reason for the decline in service footfall. Thus services should address their stance on e-cigarettes with some urgency. 

In a study of adolescent health care professionals in Minnesota (U.S.), family medicine physicians knew more about e-cigarettes and were more comfortable about talking to patients about them than nurses [28] implying again that higher status professionals might be more positive; however paediatricians had similar ratings to nurses which might suggest varying interests and networks of different groups could play a role.

### 4.1. Limitations

High quit rates from e-cigarettes uncovered from the monitoring data could be because e-cigarettes were more likely to be encouraged by services with higher quit rates generally or because of self-selection bias. Asking about e-cigarettes was a new question on monitoring returns so it was possible that use was under reported.

The online survey was very simple which meant some types of differentiation were not possible. For example, only cases with an IP address outside England or who commented that they were based elsewhere could be excluded. Thus some respondents who worked outside England could have been included if they picked a random English region (which would also reduce the accuracy of the region variable). Additionally we were unable to differentiate respondents who worked only for the SSS from personnel who were employed in another role (for example a health care assistant for a GP practice or pharmacy) which involved some smoking cessation behavioural support work. 

A different issue is that we do not know the source of SSS personnel’s opinions—we do not know the extent to which respondents’ ideas were from personal experience with clients or from general media for example. Additionally, it is unclear the extent to which the gap between managers/commissioners and practitioners is the result of practitioners having less understanding of the scientific literature on e-cigarettes or practitioners having a better understanding because they see clients who vape day to day—and thus whether it is managers/commissioners or practitioners who are more “correct” or closer to “the truth” in their beliefs.

The data analysis in this article was cross-sectional rather than longitudinal. Tracking changes over time would be useful in future work.

### 4.2. Summary

Despite a positive approach being taken towards e-cigarettes by Public Health England [4] and the NCSCT [12], in 2014 their use was not being encouraged by many English SSS and use was only recorded by a small proportion of clients. SSS commissioners and managers need to consider how to disseminate messages about e-cigarettes effectively to all practitioners and to address practitioners’ concerns.
